# Comparison of topical 0.03% tacrolimus and homologous injectable platelet-rich plasma in the treatment of keratoconjunctivitis sicca in dogs

**DOI:** 10.14202/vetworld.2023.134-143

**Published:** 2023-01-21

**Authors:** Giovana José Garcia Estanho, João Victor Goulart Consoni Passareli, Letícia da Silva Pando, Daniel Espinhosa Vieira, Gisele Alborghetti Nai, Cecília Laposy Santarém, Silvia Franco Andrade

**Affiliations:** 1 Post-graduate Program in Animal Science, UNOESTE, Presidente Prudente, São Paulo, Brazil; 2 Faculty of Veterinary Medicine, UNOESTE, Presidente Prudente, São Paulo, Brazil; 3 Department of Pathology, Faculty of Medicine, UNOESTE, Presidente Prudente, São Paulo, Brazil; 4 Department of Veterinary Ophthalmology, Veterinary Hospital, UNOESTE, Presidente Prudente, São Paulo, Brazil

**Keywords:** dogs, homologous platelet-rich plasma, keratoconjunctivitis sicca, tacrolimus 0.03%

## Abstract

**Background and Aim:**

Keratoconjunctivitis sicca (KCS) is predominantly an immune-mediated chronic inflammatory ocular disease that is commonly diagnosed in dogs. This study aimed to compare the conventional use of topical immunosuppressant tacrolimus 0.03% eye drops and a new therapy injectable homologous platelet-rich plasma (HPRP) into the third eyelid gland and inferior and superior palpebral conjunctiva of dogs with KCS.

**Materials and Methods:**

A total of 66 eyes from 33 dogs were evaluated. The eyes were divided into three equal groups: Negative control group, tacrolimus group (TG), and homologous platelet-rich plasma group (HPRPG). The animals were evaluated using the Schirmer’s tear test-1 (STT-1), osmolarity test (OT), strip meniscometry test (SMT), tear film break-up test (TBUT), fluorescein test, lissamine green test (LGT), and cytological and histopathological analyses.

**Results:**

In TG, there was a significant increase (p < 0.05) in the STT-1 and SMT values, and goblet cell count in the palpebral conjunctiva by the end of the study. In HPRPG, 36% (four dogs) received three applications, 55% (six dogs) received two applications, and 9% (one dog) received one application before the initial ocular signs improved. There was a significant decrease (p < 0.05) in the lymphocyte and neutrophil counts of the palpebral conjunctiva in HPRPG than in TG. Both groups showed equivalent improvements in TBUT, OT, and LGT values.

**Conclusion:**

Tacrolimus 0.03% eye drops were more efficient than HPRP in increasing tear production and the number of goblet cells. However, injectable HPRP was more efficient than tacrolimus in decreasing the number of conjunctival inflammatory cells. Treatment with injectable HPRP requires an average of two to three applications, is safe and feasible, and can be used as a cheaper alternative or as an adjuvant to conventional treatment with topical immunosuppressants.

## Introduction

Keratoconjunctivitis sicca (KCS), also known as dry eye disease, is characterized by chronic inflammation of the tear glands, causes decreased tear production (quantitative KCS) or deficiency in tear quality (qualitative KCS), or both. Immune-mediated KCS is the most common type in dogs and humans [1–3]. The main clinical signs of KCS include loss of brightness and transparency of the cornea, conjunctival hyperemia, vascularization, and the presence of mucous or mucopurulent secretions. In severe cases, the disease can cause vision loss due to loss of corneal transparency or perforations [4, 5].

The following tests are used in the diagnosis of KCS: Schirmer’s tear test (STT-1), tear film break-up test (TBUT), tear osmolarity test (TOT), Lissamine Green test (LGT), Rose Bengal test, phenol red test, evaluation of the meibomian glands and tear film, cytological exam, and histopathological exam of the conjunctiva [6]. Schirmer’s tear test-1 is a routinely used quantitative diagnostic test for KCS. The strip meniscometry test (SMT) is used to assess the tear quantity in the lacrimal meniscus [6–8]. Tear film break-up test is a qualitative clinical test used to assess evaporative dry eye disease; TOT is a qualitative marker of disease severity but, with higher sensitivity and specificity than TBUT [9, 10]. Keratoconjunctivitis sicca causes tear film instability resulting in tear hyperosmolarity [11].

Conventional treatment includes a combination of topical drugs, artificial tears or ocular lubricants, and calcineurin inhibitors that have tear-stimulating and immunosuppressive effects, such as tacrolimus, cyclosporin A, and pimecrolimus. These drugs suppress interleukin-2 production, prevent the formation of inflammatory cascade and eosinophil recruitment, and reduce inflammation by inhibiting histamine release and prostaglandin production [12–15]. Tacrolimus is a lipophilic macrolide antibiotic isolated from *Streptomyces tsukubaensis* spp. that causes the proliferation of goblet cells. It inhibits the immune response by binding to FK506-binding protein, which impedes the initial phase of type-T lymphocytes activation. The intraocular action of tacrolimus can be variable and potentiated according to the concentration and transport vehicle used [13–17].

Platelet-rich plasma (PRP), a byproduct derived from blood plasma, has tissue-reconstituting and reparative properties since it contains growth factors, cytokines, integrins, and Vitamin A. Platelet-rich plasma is rich in bioactive proteins synthesized in the blood, mainly platelet alpha granules. It has an immunomodulatory function, can be easily obtained and used, and is inexpensive [18]. In patients with dry eye disease, treatment with injectable PRP is an effective therapy that promotes increased tear production, provides better results on TBUT, improves visual acuity, and decreases signs of dry eye [19–21].

Platelet-rich plasma (can be autologous (extracted from the individual), homologous (extracted from the same species), or heterologous (extracted from another species) in origin. Homologous and heterologous forms of PRP are only used when autologous PRP is unavailable. First, the whole blood of the donor is centrifuged, with the addition of anticoagulants such as thrombin or sodium citrate. Sedimentation occurs during this pro­cess, leaving the platelets suspended in the plasma at a concentrated level; the growth factors are then released. The process of obtaining the PRP takes an average of 1 h. The platelet concentration in PRP is usually 4–7 times higher than that in whole blood [18–21].

This study aimed to evaluate and compare two treatment methods for KCS: A new treatment by injecting homologous PRP (HPRP) into the third eyelid gland, and lower and upper conjunctiva versus conventional treatment with tacrolimus 0.03% eye drops, a topical immunosuppressant.

## Materials and Methods

### Ethical approval

The study was approved by the Ethical Committee on Animal Use of Universidade do Oeste Paulista (UNOESTE - Protocol No. 5726) and was conducted according to the Association for Research in Vision and Ophthalmology guidelines for the use of animals in ophthalmic and visual research.

### Study period and location

The study was conducted from August 2019 to July 2021 in Veterinary Hospital (VH) and kennel at UNOESTE, Presidente Prudente, São Paulo, Brazil.

### Animals and study design

Dogs diagnosed with KCS were identified during routine clinical visits to the ophthalmology service of the VH of the UNOESTE. The dogs in the negative control group (NCG) were selected from the kennel at UNOESTE. A total of 33 dogs (66 eyes) of both sexes and different breeds, aged 7.5 ± 3.6 (1.0–15.0) years and weighing 11.7 ± 10.5 (3.6–43) kg, were included in the study. The animals were divided into three groups (NCG and two KCS treatment groups). The inclusion criteria for the study were as follows: For healthy eyes – rate of lacrimation on STT-1 without anesthetic ≥15 mm/min, TBUT of >20 s, and no remarkable findings on ophthalmic examinations; and for eyes with KCS – presence of ophthalmic clinical signs (ocular secretion, conjunctival hyperemia, opacity, corneal neovascularization, and corneal pigmentation), lacrimation rate on STT-1 <15 mm/min or TBUT <20 s, or both. The guardians of each animal signed an informed consent form. All ocular tests and examinations were performed by the same examiner (GJGE).

### Groups

Three groups of dogs, each with 11 animals (22 eyes), were evaluated: the NCG contained dogs who did not have KCS; dogs that were diagnosed with KCS were randomly assigned to either the tacrolimus group (TG) or homologous PRP group (HPRPG). The characteristics of the groups are described below.

Negative control group (n = 22 normal eyes): 11 healthy dogs (six males and five females); aged 4.3 ± 1.9 (1.0–7.0) years; weighing 20.4 ± 14.9 (5.0–43.0) kg; nine mixed-breed dogs, one Boxer, and one Labrador. The NCG was used to extract reference ocular values for the study region and to serve as a comparison group with the KCS-positive groups.

Tacrolimus group (n = 22 eyes positive for KCS): 11 dogs (five males and six females); aged 8.5 ± 2.1 (5.0–11.0) years; weighing 7.5 ± 1.4 (5.2–9.8) kg; seven Lhasa Apso, one Shih Tzu, one Yorkshire Terrier, and one mixed-breed dog. Once the diagnosis of bilateral KCS was confirmed, the following protocol was established: topical treatment with 0.03% tacrolimus eye drops (Laboratory Centro Paulista, São Paulo, SP), at one drop twice a day in both eyes, and eye lubricant based on 20% chondroitin A sulfate (Laboratory Labyes, Valinhos, São Paulo, Brazil), at one drop twice a day, in both eyes, for 6 months.

**Figure-1:**
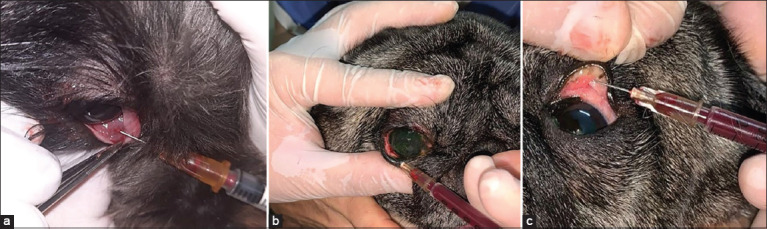
Homologous platelet-rich plasma application points (a) gland of the third eyelid, (b) inferior eyelid conjunctiva, and (c) superior eyelid conjunctiva.

Homologous platelet-rich plasma group (n = 22 eyes positive for KCS): 11 dogs (four males and seven females); aged 9.7 ± 4.0 (5.0–15.0) years; weighing 7.1 ± 2.4 (3.6–12.1) kg; six Lhasa Apso, two Shih Tzu, two Yorkshire Terriers, one Cocker Spaniel, and one mixed-breed dog. Once the diagnosis of bilateral KCS was confirmed, the following protocol was established: In the ambulatory clinic, one drop of anesthetic eye drops (Allergan, São Paulo, Brazil) was instilled in each eye topically, 3 times at an interval of 5 min; then, 0.3 mL of HPRP was administered with an insulin syringe and needle at 0.1 mL in the third eyelid gland, 0.1 mL in the inferior palpebral conjunctiva, and 0.1 mL in the superior palpebral conjunctiva of both eyes (Figure-1). In addition, a topical ocular lubricant (Laboratory Labyes) was prescribed, at one drop twice a day in both eyes, for 6 months. The frequency of injectable HPRP administration was based on the initial improvement of clinical signs and the STT-1 and TBUT results for a maximum of three applications. The dogs whose condition did not improve after the third application were switched to conventional treatment with topical immunosuppressants.

If diagnosed with secondary bacterial infection and ocular inflammation (conjunctivitis or keratitis) in TG and HPRPG, ciprofloxacin-based antibiotic eye drops (Laboratory Labyes), at one drop 3–4 times a day and sodium diclofenac-based anti-inflammatory eye drops (Allergan), at one drop 3 times a day for 15 days, were prescribed [17, 22, 23].

### Homologous platelet-rich plasma processing

Homologous platelet-rich plasma was prepared at the clinical pathology laboratory of the VH of UNOESTE. Only one healthy 5-year-old (weight 35 kg) of mixed-breed was used as the donor dog. In detail, 20 mL of whole blood was collected in sterile tubes containing 3.2% sodium citrate using the vacuum system for each animal that received therapy by puncturing the jugular vein. In the first stage, the blood was centrifuged at 200 RCF or 1,100 RPM for 10 min in a laboratory centrifuge (Excelsa, FANEM, São Paulo, Brazil). Approximately 30% of the surface plasma was discarded, and the remaining content was transferred to a dry and sterile Falcon tube, which was then centrifuged at 400 RCF or 1900 RPM for 10 min. At this stage, the plasma was divided into two portions, PRP and platelet-poor plasma. Two-thirds of the supernatant material was discarded (platelet-poor plasma), and the remaining one third was classified as PRP. A total volume of 0.6 mL was obtained from this material, and 0.1 mL was instantly applied to the third eyelid gland, and lower and upper eyelid conjunctiva (total application of 0.3 mL/eye).

### Evaluation of clinical signs

Ophthalmic signs were evaluated monthly during 6 months of treatment, from moment M0 (time of diagnosis of KCS) to M6 (final treatment and evaluation of KCS). The ophthalmic signs of each animal were evaluated using a portable slit lamp (SL-15, Kowa, Japan); a specific form was used to evaluate corneal opacity, pigmentation, neovascularization, presence of ocular secretion, and conjunctival hyperemia. The following scoring system was adopted for the assessment: (0) no, (1) light, (2) moderate, and (3) severe change.

### Ophthalmic tests

Specific tests were performed at all follow-up time points. The following ophthalmic tests were adopted in the given sequence: TOT, STT-1, SMT, TBUT, fluorescein test (FT), and LGT.

Tear osmolarity test was measured using an I-PEN VET osmometer (I-Med Pharma Inc., USA). The disposable sensor chip of the device placed in contact with the conjunctiva at an angle of 45° measured the concentration of the tear solute, indicating the presence of tear film instability and aiding the qualitative diagnosis of KCS. The reference osmolarity values ranged from 296 mOsmol/L to 339 mOsmol/L [24].

Schirmer’s tear test-1, a quantitative tear test was performed without local anesthetic. The eyes were cleaned with dry cotton and the tip of filter paper (0.5 cm long) was placed into the conjunctival sac (Schirmer test, Ophthalmos, São Paulo, Brazil) for 1 min. Once removed, a reading was measured immediately according to the moistened length of the filter paper. Eyes with STT-1 values <15 mm/min were considered positive for KCS [7].

Strip meniscometry test, quantitative tear test similar to the STT was performed using a disposable paper strip (I-Tear, I-MED, Canada) with a central column and scale printed on both sides. The strip was gently placed against the tear meniscus of each eye for a period of 5 s and the length of the central column stained blue by the tears was recorded. Values from 0 mm to 7 mm/5 s were considered positive for KCS [8].

Tear film break-up test, a qualitative tear test, was performed by administering one drop of 1% fluorescein eye drops (Ophthalmos) on the cornea. After two blinks, the eyelid was held open manually, and the cornea was observed with a portable slit lamp (SL-15) with cobalt blue illumination, and with the assistance of a stopwatch, the tear film break-up time (appearance of the first dry spots on the cornea) was measured in seconds. Two repetitions were performed, and the average was calculated. Tear film break-up test values ≤20 s were considered positive for KCS [25].

Fluorescein test was performed using a vital dye fluorescein that stains the corneal stroma [7]. One drop of 1% fluorescein eye drops (Sodium Fluorescein, Ophthalmos) was instilled on the cornea, and the eye was washed with saline solution and examined using a portable slit lamp to check for a corneal ulcer. Scores, 0 (negative) and 1 (positive) were used for classification.

Lissamine green test was performed using a vital dye, lissamine green, that stains devitalized cells in the cornea and ocular conjunctiva due to the inefficiency of tear protection caused by KCS [7]. This test was performed using sterile strips containing lissamine (Ophthalmos) soaked in one drop of saline solution. The strip was then placed against the conjunctival sac and the eyes were examined with a portable slit lamp regardless of staining. Scores, 0 (negative) and 1 (positive) were used for classification.

### Cytological and histopathological analysis

Cytological analysis of third eyelid gland was performed at M0, M3, and M6, and samples were collected by fine-needle aspiration. To assemble the samples, one drop of anesthetic eye drops (1% tetracaine hydrochloride + 0.1% phenylephrine hydrochloride, Allergan) was instilled to desensitize the glands of the collected eye; after exposing the third eyelid gland using tweezers, samples of the glands were collected, and one drop each of adrenaline and diclofenac sodium (Allergan) was instilled; finally, methanol and the May-Grunwald-Giemsa stain were used to fix the samples. The numbers of neutrophils, lymphocytes, and squamous cells in the epithelium were evaluated. Cell counts on each slide were performed in 10 high-power fields using an optical microscope at 400× magnification (Nikon Eclipse E200, Tokyo, Japan).

Histopathological analysis of the conjunctiva was performed at M0 and M6 of the study. A drop of anesthetic eye drops (1% tetracaine hydrochloride + 0.1% phenylephrine hydrochloride, Allergan) was instilled in each eye, and cuts were made using conjunctival scissors approximately 1.0 mm from the inferior bulbar conjunctiva and placed on a piece of paper (1 × 1 cm). Subsequently, one drop of adrenaline and one drop of diclofenac sodium (Allergan) were instilled in the conjunctiva. The collected fragments were fixed in formalin and stained using hematoxylin-eosin (HE) (Dolles, São Paulo, Brazil) and periodic acid-Schiff (PAS) (Merck, USA) techniques. The HE-stained samples were used to evaluate the numbers of neutrophils and lymphocytes and the presence of metaplasia; the PAS technique was used to count the goblet cells. Cell count on each slide was performed in four different fields using a 400× magnification (Nikon Eclipse E200); Leica ICC50HD (Wetzlar, Germany) was used for image recording.

### Statistical analysis

Bidirectional analysis of variance (two-way analysis of variance) for pairs of samples with Tukey’s *post hoc* test was used to analyze the data from the STT-1, SMT, TBUT, TOT, and cytology and histopathological report. Friedman non-parametric test was used to compare the results of FT and LGT variables at various time points. The Kruskal–Wallis test with Dunn’s *post hoc* test was used to compare variables between groups. Statistical significance was set at p < 0.05. All analyses were performed using R version 4.1.3 (R Development Core Team, 2022).

## Results

The ophthalmic, cytological, and histopathological parameters of healthy dogs in the NCG are summarized in Table-1. The initial mean platelet count in the blood collected for HPRP preparation was 193,643 (70,000–257,000)/mm^3^; and after HPRP preparation, the final mean platelet count was 1,997,143/mm^3^ (1,040,000–4,297,000)/mm^3^.

**Table-1:** Parameters of the negative control group (NCG) (22 healthy eyes; 11 healthy dogs) Mean and standard deviation (minimum and maximum) values of the parameters: Schirmer’s tear test-1 (STT-1), SMT, tear film break-up test (TBUT), and TOT. Mean and standard deviation of the third eyelid gland cytology and inferior bulbar conjunctival histopathology.

Ophthalmic tests	Mean and standard deviation	Minimum and maximum
STT-1 (mm/min)	22.3 ± 2.9	(18 – 27)
SMT (mm/5 sec)	10.4 ± 3.3	(8 – 18)
TBUT (sec)	26.8 ± 4.9	(20 – 37)
OTT (mOsmol/L)	299.2 ± 13.9	(288 – 311)

**Cells**	**Third eyelid gland cytology (Mean and standard deviation)**	**Histopathological analysis of the conjunctiva (Mean and standard deviation)**

Neutrophils	0 ± 0	02.6 ± 04.4
Lymphocytes	0.1 ± 0.4	26.7 ± 24.2
Goblet cells	N.E*	28.3 ± 16.0
Squamous cells	0.1 ± 0.3	N.E*

* N.E: not evaluated.

The required number of applications that improved the initial ocular signs in HPRPG (n = 11 dogs/n = 22 eyes) were as follows: Four dogs/eight eyes (36% cases) received three applications, six dogs/twelve eyes (55% cases) received two applications, and one dog/two eyes (9%) received one application. Of all eyes (n = 22 eyes) that received HPRP application, only one eye (4.5%) had a mild inflammatory reaction to the blood product on the day of application, which regressed after treatment with anti-inflammatory eye drops (diclofenac eye drops, one drop 3 times/day, for 3 days). None of the dogs in HPRPG had to withdraw from the study. In TG, one dog/both eyes had a severe inflammatory reaction to the tacrolimus eye drops on the 1^st^ day of treatment, making daily instillation impossible. Therefore, the dog was removed from the study and replaced by another with KCS. The dog with the adverse reaction was referred to the VH ophthalmology service, and the use of cyclosporine eye drops was recommended.

Both treatment groups showed improvements in the clinical signs and complete remission of signs at M5 (Table-2). Opacification, secretion, and hyperemia in HPRPG had regressed at M5. In TG, secretion and opacification showed remission at M3, while pigmentation and conjunctivitis showed complete resolution at M1. In this group, neovascularization had remitted at M5 of treatment.

**Table-2:** Evaluation scores of the median ocular signs of homologous platelet-rich plasma group (HPRPG) and tacrolimus group (TG), treatment groups at the start (M0) and every 30 days from moment one (M1) to moment six (M6).

Ocular signs	M0	M1	M2	M3	M4	M5	M6
Corneal pigmentation HPRPG	1	1	0	0	0	0	0
TG	1	1	0	0	0	0	0
Corneal neovascularization HPRPG	1	1	1	1	1	0	0
TG	1	1	1	1	1	0	0
Ocular secretion HPRPG	3	1	1	1	1	0	0
TG	3	1	1	0	0	0	0
Corneal opacity HPRPG	1	1	1	1	1	0	0
TG	1	1	1	0	0	0	0
Conjunctival hyperemia HPRPG	2	1	0	0	1	0	0
TG	2	1	0	0	0	0	0

**Figure-2:**
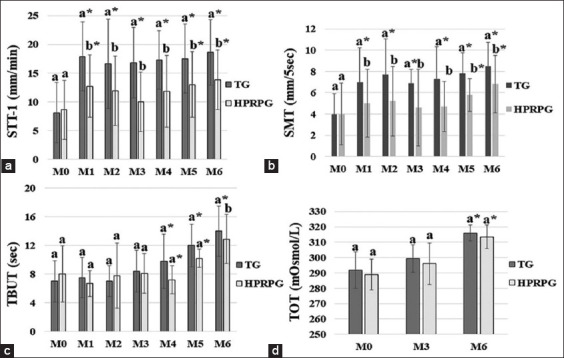
Means and standard deviation of eye test results: (a) Schirmer tear test-1 (STT-1) in mm/min, (b) strip meniscometry test (SMT) in mm/5 s, (c) tear film break-up test (TBUT) in seconds, and (d) tear osmolarity test (TOT) in mOsmol/L for tacrolimus group (TG) and homologous platelet-rich plasma group (HPRPG) at the start (M0) and at every 30 days from moment one (M1) to moment six (M6).

A significant increase (p < 0.05) in the values of STT-1 (Figure-2a) and SMT (Figure-2b results was observed from M0 to M6; TG showed better improvement than HPRPG at M6. The TBUT (Figure-2c) and TOT (Figure-2d) results showed a significant increase (p < 0.05) from M0 to M6, with no significant differences (p > 0.05) at M6 between the groups.

**Figure-3:**
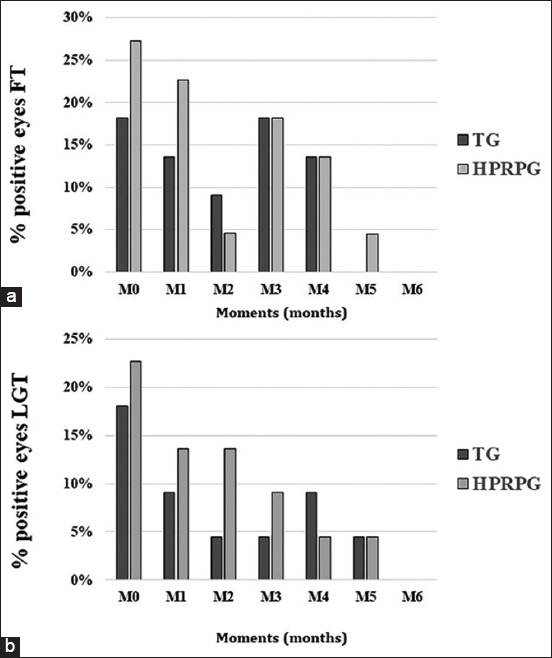
Percentage of stained eyes: (a) Fluorescein test, and (b) lissamine green test, in dogs with keratoconjunctivitis sicca (n = 22) in tacrolimus group (TG) and homologous platelet-rich plasma group (HPRP) at the start (M0), and every 30 days from moment one (M1) to moment six (M6).

The FT results are depicted in Figure-3a and LGT results are in Figure-3b. There were negative markings of the FT in TG at M5 and in HPRPG at M6. For the LGT, both groups showed similar results at M5 (TG) and M6 (HPRPG).

**Figure-4:**
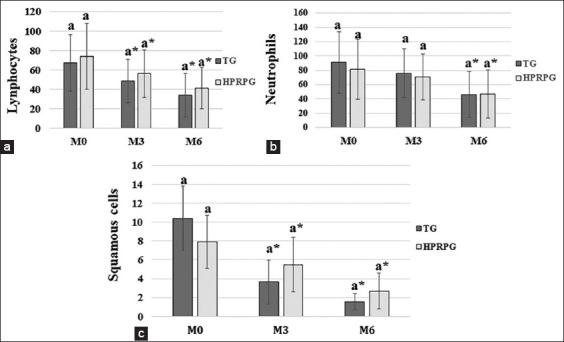
Means and standard deviations of the counts of (a) lymphocytes, (b) neutrophils, and (c) squamous cells, in the cytological examination of the third eyelid gland performed at M0 (start), M3 (3 months), and M6 (6 months).

The cytology results of the third eyelid gland (neutrophils, lymphocytes, and squamous cells) are shown in Figure-4. A significant decrease (p < 0.05) in the number of neutrophils, lymphocytes, and squamous cells in both groups was observed at the end of the study (M6), with no statistically significant difference (p > 0.05) between the groups.

**Figure-5:**
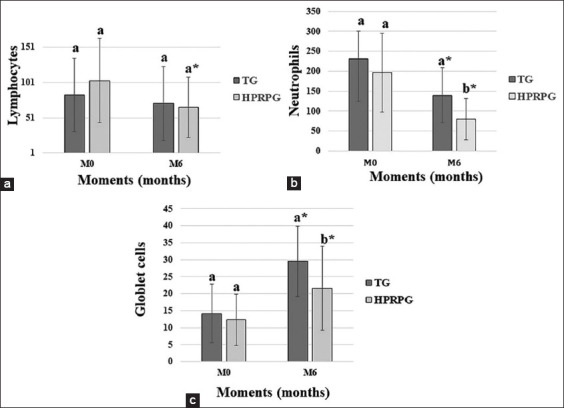
Means and standard deviations of the counts of (a) neutrophils, (b) lymphocytes, and (c) goblet cells in the histopathological examination of the inferior bulbar conjunctiva, performed at M0 (start) and M6 (6 months).

**Figure-6:**
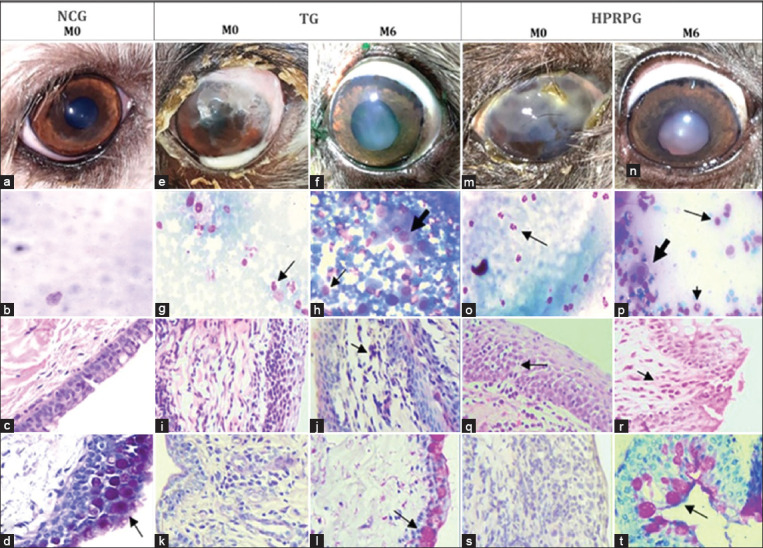
Comparison of the negative control group (NCG) with the clinical evolution of tacrolimus group (TG) and homologous platelet-rich plasma group (HPRP) at the start (M0) and end (M6). NCG - right eye: (a) no change; (b) cytology of the third eyelid gland of the eye without keratoconjunctivitis sicca (KCS); (c) conjunctival histopathology showing unaltered epithelium; (d) conjunctival histopathological examination showing a large number of goblet cells (arrow); TG - left eye: (e) M0, mucopurulent secretion and corneal vascularization; (f) M6, clinical evolution and improvement in KCS signs; (g) M0, third eyelid gland cytology showing the presence of neutrophils (arrow); (h) M6, third eyelid gland cytology with the presence of goblet cells (thick arrow) and lymphocytes (thin arrow); (i) M0, conjunctival histology with lymphocytes in the stroma and epithelial edema; (j) M6, conjunctival histology with rare lymphocytes in the stroma (arrow); (k) M0 histology with absence of goblet cells; (l) M6 histology showing the presence of normal goblet cells (arrow); HPRP group - right eye: (m) M0, mucopurulent secretion and corneal pigmentation; (n) M6, clinical evolution and improvement in the signs of KCS; (o) cytology of the third eyelid gland with the presence of neutrophils (arrow) in the mucus; (p) M6, third eyelid gland cytology with the presence of goblet cells (thick arrow), neutrophils (arrowhead) and lymphocytes (thin arrow); (q) M0, histology showing chronic inflammation with conjunctival edema and lymphocyte exocytosis (arrow); (r) M6, conjunctival histology showing the presence of rare lymphocytes in the stroma (arrow); (s) M0, histology showing the absence of goblet cells; (t) M6, histology showing the presence of normal goblet cells (arrow); In b, g, h, o, and p: staining and Giemsa, 400x magnification; in c, i, j, q, and r: hematoxylin-eosin staining, 400× magnification; in d, k, l, s, and t: PAS staining, 400× magnification.

The histopathological examination results of the conjunctiva are shown in Figure-5. There was a decrease in the number of lymphocytes and neutrophils at M6 in both groups, with a more significant (p < 0.05) reduction in HPRPG than in TG. There was a significant increase in the number of goblet cells (p < 0.05) in both groups, with a statistically significant high cell count in TG. There was a decrease in the presence of metaplastic cells in both groups at M6, although it was more pronounced in TG (45% at M0, 9% at M6) than in HPRPG (27% at M0, 14% at M6). However, there was no statistically significant difference in the frequency of metaplastic cells in both groups (p > 0.05). The comparisons of the NCG with TG and HPRP at M0 and M6 are shown in Figure-6.

## Discussion

This is the first study to compare the use of injectable HPRP in ambulatory care for dogs diagnosed with KCS with that of conventional topical treatment using tacrolimus 0.03% eye drops. The use of HPRP in ambulatory care requires practice and synchronization of the time between preparation and application, as the blood product must be applied instantly [26]. Application methods adjacent to the eyes can cause momentary local discomfort to the patient even when using anesthetic eye drops and containment; therefore, applications were performed at an interval of 30 days.

In this study, only one eye of a dog developed a local hypersensitivity reaction caused by the application of HPRP, which was quickly treated and eventually controlled. Platelet-rich plasma from homologous and heterologous sources can cause adverse reactions owing to immunogenic differences between individual organisms. Adverse reactions to the use of autologous PRP may occur because of the substance added to the PRP preparation [27, 28]. A blood compatibility test does not identify the risk of incompatibility of the recipient for therapy with PRP from a homologous or heterologous source, as the test only identifies the presence of antibodies in the recipient’s serum against the donor’s red cells [28]. Adverse signs of topical use of tacrolimus include eye irritation, stinging, redness, and burning sensation, which are often transitory signs. The same was observed in our study, where one dog developed an adverse reaction to the medication, which was promptly treated, and the dog was removed from the study [29].

Subconjunctival application is an effective route to replace the daily instillation of eye drops [30]. The application of autologous PRP through this route in dogs with moderate KCS was also studied by Vatnikov *et al*. [21], who carried out weekly PRP applications only to the lower eyelid conjunctiva. The inferior and superior subconjunctival spaces were used in our study to stimulate the meibomian glands and goblet cells, responsible for producing tear lipids and mucin, respectively [11]. Furthermore, the choice of PRP application to the upper eyelid conjunctiva was based on its proximity to the lacrimal gland.

The average weight of dogs treated with PRP was <10 kg (Table-2), and some animals weighed <5 kg, which would make it difficult to use PRP of autologous origin. The effectiveness of HPRP is equivalent to that of autologous tissue [31–33]. In our study, the process of obtaining HPRP enriched the average platelet concentration to above one million platelets and is consistent with that reported in other studies. On an average, platelet count is five times higher than the basal blood after enrichment; this is necessary to ensure the effectiveness of PRP [26, 32].

There were improvements in the clinical signs related to the ocular surface in both groups, such as decreased hyperemia, conjunctival secretion, opacity, and corneal pigmentation. Overall, TG exhibited earlier signs of remission than HPRPG. Some clinical signs showed early improvement in TG, while others stabilized only at M5. Radziejewski and Balicki [15] observed complete remission of all clinical signs after 2 months of treatment with tacrolimus. The clinical signs of pigmentation and hyperemia were the first to improve. Berdoulay *et al*. [34] reported improvements in neovascularization and opacification just 15 days after the start of treatment. Whereas in our study, these phenomena only occurred after 4 months of treatment.

Based on STT-1, the use of topical 0.03% tacrolimus had normalized tear production (≥15 mm/min) after the 1^st^ month of treatment and its effectiveness remained superior to HPRP throughout the study. On the SMT, despite the increase in tear production, the groups did not reach average normality. By the end of the study, HPRPG and TG showed differences in STT-1 and SMT results, but TG obtained mean STT-1 values similar to that of the NCG. The dogs in HPRPG received a maximum of three PRP applications, in contrast to the group treated with 0.03% tacrolimus that received daily instillation of eye drops twice a day throughout the study.

The results in HPRPG were apparent after the 1^st^ month of treatment and were consistent with the results of a study involving humans, conducted by Avila *et al*. [20], who observed that injectable PRP treatment for humans with severe dry eye disease was effective at 4 weeks after the first application. The STT-1 and SMT results showed improvements soon after the start of treatment in our study.

In a study of dogs with healthy eyes, Lamkin *et al*. [24] used the I-PEN VET osmometer, same as the one used in our study and reported an average baseline normality was 318.55 ± 20.82, in contrast to that observed in the NCG in our study; a slight increase in osmolarity was observed in the treatment groups, although within the reference values. The literature highlights that reflex tearing can cause a decrease in tear osmolarity [24–35]; thus, the TOT was the first test in the sequence adopted in the present study. Nolfi and Caffery [36] evaluated I-Pen and TearLab osmometers and reported considerable variations between the two; I-Pen was more sensitive to manipulation and showing greater variations in its results. In our study, due care was taken to avoid variations, and only one evaluator performed the tests with these devices.

Ulcerative lesions were present in both groups at the time of diagnosis and were present up to M4 in TG and M5 in HPRPG. This result can be attributed to the fact that most treated dogs were brachycephalic dog breeds (Lhasa Apso and Shih Tzu). Their eyes are more exposed to the environment and susceptible to trauma due to their orbital structure and greater ocular prominence [36, 37]. Farghali *et al*. [38] reported that the use of subconjunctival injection of autologous PRP was an effective treatment for corneal ulcers in dogs and cats, and the number of injections was dependent on the breed type.

There was a considerable decrease in the total number of inflammatory cells in both groups, which was attributed to the immunomodulatory effects of the agents studied. However, the numbers of neutrophils, lymphocytes, and squamous cells in the treatment groups at the end of the study did not match the same parameters as those in the NCG. This can be explained by the chronic and progressive nature of the inflammatory process in KCS, where the objective of treatment is to reduce inflammation and improve clinical signs of the disease [15].

In the cytopathological analysis, more neutrophils than lymphocytes were initially observed. At the final time point (M6), the neutrophil count in TG was significantly lower than in HPRPG. This can be attributed to the immunosuppressive potential of tacrolimus, which acts by calcineurin inhibition and T lymphocyte activation, thus reducing the inflammatory response [15]. Histopathological analysis showed that HPRPG showed a greater reduction in the inflammatory and infectious process than TG. According to Badade *et al*. [39], the concentrated plasma in PRP has a two-to-four-fold higher concentration of leukocytes than whole blood and thus can generate neutrophils, lymphocytes, and monocytes. They have a bacteriostatic function and defense against fungi and other microorganisms by releasing cytokines and defense cells [39, 40].

There was also an increase in the number of goblet cells in TG and HPRPG at the end of the study. The use of tacrolimus resulted in a higher density of goblet cells in TG than in HPRPG and NCG. The action of tacrolimus on mucin-producing cells was also observed in other reports [17, 34]. In a study by Alio *et al*. [18], the density of goblet cells had significantly increased in the eyes treated with PRP; despite the lack of statistical significance in our study, there was an increase in the number of goblet cells in HPRPG at M6 compared to that at M0.

## Conclusion

Tacrolimus 0.03% eye drop was found to be more efficient than HPRP in increasing tear production and the number of goblet cells. However, injectable HPRP was more efficient than tacrolimus in decreasing conjunctival inflammatory cells. Treatment with injectable HPRP requires an average of two to three applications, is safe and feasible, and can be used as a cheaper alternative or as an adjuvant to conventional treatment with topical immunosuppressants. For dogs with KCS whose owners experience difficulty in the daily use of topical eye drops or find the cost of these eye drops to be a limiting factor in the treatment, the use of injectable HPRP can be an interesting alternative.

## Authors’ Contributions

GJGE: Designed and conducted the experiment, performed all animal examinations and tests, analyzed the data, prepared the graphs, figures, and tables, and drafted the manuscript. JVGCP, LSP, and DEV: Performed all animal examinations and tests. GAN: Performed the cytological and histopathological analyses. CLS: Conceptualized the aim of the study, planned, supervised, designed, and conducted the experiment, analyzed the data, and prepared HPRP. SFA: Conceptualized the aim of the study, planned, supervised, designed, and conducted the experiment, performed all animal examinations and tests, analyzed the data, prepared the graphs, figures, and tables, and drafted the manuscript. All authors have read and approved the final manuscript.
